# Chondrogenic Differentiation from Induced Pluripotent Stem Cells Using Non-Viral Minicircle Vectors

**DOI:** 10.3390/cells9030582

**Published:** 2020-03-01

**Authors:** Yeri Alice Rim, Yoojun Nam, Narae Park, Hyerin Jung, Kijun Lee, Jennifer Lee, Ji Hyeon Ju

**Affiliations:** 1 Catholic iPSC Research Center, College of Medicine, The Catholic University of Korea, Seoul 06591, Korea; llyerill0114@gmail.com (Y.A.R.); givingtreemax@gmail.com (Y.N.); narae5322@gmail.com (N.P.); ilovehyelin@gmail.com (H.J.);; 2 Division of Rheumatology, Department of Internal Medicine, Seoul St. Mary’s Hospital, Institute of Medical Science, College of Medicine, The Catholic University of Korea, Seoul 06591, Korea; poohish@naver.com

**Keywords:** induced pluripotent stem cell, minicircle, chondrogenesis, BMP2, TGFβ3, growth factor, transfection

## Abstract

Human degenerative cartilage has low regenerative potential. Chondrocyte transplantation offers a promising strategy for cartilage treatment and regeneration. Currently, chondrogenesis using human pluripotent stem cells (hiPSCs) is accomplished using human recombinant growth factors. Here, we differentiate hiPSCs into chondrogenic pellets using minicircle vectors. Minicircles are a non-viral gene delivery system that can produce growth factors without integration into the host genome. We generated minicircle vectors containing bone morphogenetic protein 2 (BMP2) and transforming growth factor beta 3 (TGFβ3) and delivered them to mesenchymal stem cell-like, hiPSC-derived outgrowth (OG) cells. Cell pellets generated using minicircle-transfected OG cells successfully differentiated into the chondrogenic lineage. The implanted minicircle-based chondrogenic pellets recovered the osteochondral defects in rat models. This work is a proof-of-concept study that describes the potential application of minicircle vectors in cartilage regeneration using hiPSCs.

## 1. Introduction

The poor recovery of damaged cartilage has prompted researchers to develop a defined regeneration strategy [1]. The repair technique used for articular cartilage is mostly done using various cell sources with limited propagating ability. Chondrocytes are usually obtained autologously or generated in vitro from adult stem cells (i.e., adipose-derived stem cells and mesenchymal stem cells) [2,3]. However, primary chondrocytes and adult stem cells are in short supply and easily lose their characteristics under in vitro culture conditions. It has also been reported that the chondrogenic potential of adult stem cells depends on the pathological status of the donor. Because of these reasons, studies on regeneration using pluripotent stem cells have robustly been carried out over the past decade. Human induced pluripotent stem cells (hiPSCs) have shown boundless possibilities in tissue regeneration. To this end, hiPSCs expand significantly and maintain their pluripotency for several passages [4]. The use of hiPSCs in cell-based therapy is promising for damaged tissues with low regenerative abilities.

Recombinant human growth factors are now considered to be a critical supplement in the field of tissue engineering for regenerative medicine. Current differentiation protocols of stem cells towards chondrocytes are done with several growth factors, such as bone morphogenetic proteins (BMPs) and transforming growth factors (TGFs) [5,6]. BMPs were originally known to be critical in the development of bone and cartilage [7,8]. The deletion of BMP2 results in severe defects during endochondral bone development [9]. This has revealed the role of BMP2 in chondrocyte survival and proliferation. The TGFβ superfamily of proteins control the architecture of various tissues by contributing to processes such as proliferation, differentiation, and apoptosis. Between all TGFβ proteins, three isoforms (i.e., TGFβ1, TGFβ2, and TGFβ3) are reported to induce chondrogenesis [10,11,12,13,14]. Most research has been done with TGFβ1 or TGFβ3 [15]. TGFβ3 is reported to have a higher differentiation ability than TGFβ1 [16]. However, it was also reported that the isotype is not a critical issue after a certain time point [15]. Combining several growth factors for chondrogenesis was reported to have an enhancing effect on the differentiation process. The combination of BMP2 and TGFβ3 in a three-dimensional culture system enhanced the chondrogenic differentiation of bone marrow-derived mesenchymal stem cells (MSCs) [17]. However, the frequent addition of growth factor molecules during differentiation is cost-effective. The overexpression of several growth factors by gene delivery is efficient in chondrogenesis. Retroviral delivery of TGFβ1 in synovial-derived MSCs enhances the proliferation of cells and accelerates chondrogenic differentiation [18]. The overexpression of SOX9 enhances differentiation in mouse MSCs and umbilical cord blood-derived MSCs [19,20]. Increased collagen type II expression has been confirmed in mouse embryonic stem cells after human SOX9 overexpression [21].

Non-viral gene delivery is promising for use as a safe in vivo gene transfer strategy. Yet, commercial plasmid DNA vectors contain bacterial sequences that may induce immune responses by producing antibodies against bacterial proteins [22]. Gene expression in the host cell can be altered by antibiotic resistance markers and immune responses [23]. Minicircles are vectors with eliminated bacterial backbones and transcription units, including the antibiotic resistance gene. Therefore, they have a relatively small size compared to other commercial vectors. The small size and the ability to avoid immune reactions leads to the high expression of the foreign gene, both in vitro and in vivo. Minicircles also show potential in pre-clinical gene therapy research [24].

Safe and efficient gene transfer is promising for the use of gene-modified stem cells in therapeutic applications [25]. The effect of minicircle vectors encoding human proteins has been confirmed through our preliminary research studies [26,27,28]. The combination of minicircle vectors and stem cells can suggest a new regenerative tool for clinical applications. In this study, we differentiated hiPSCs into chondrogenic pellets using minicircles encoding the cDNA of BMP2 and TGFβ3. Outgrowth (OG) cells were induced from hiPSC-derived embryoid bodies (EBs). Minicircles were transfected in hiPSC-derived OGs. The transfected OG cells were generated into chondrogenic pellets and maintained for 30 days. The chondrogenic pellets have further been characterized using various assays. Minicircle-induced chondrogenesis using hiPSCs suggests a new approach for future applications in tissue engineering and regenerative medicine.

## 2. Materials and Methods

### 2.1. Minicircle Production

The parental plasmid (an expression cassette consisting of CMV-MCS-EF1-RFP-SV40-PolyA with antibiotics, the kanamycin resistance gene, and the replication the pUC Ori cassette, flanked by the attP and attB sites, which are recognized by φC31 intergrase) was purchased from SBI (System Biosciences, Mountain View, CA, USA). The cDNA sequence of a codon-optimized human BMP2 and TGFβ3 was subcloned into the mock parental plasmid. The growth factor cDNA was inserted at the BamHI and XbaI restriction sites in the multiple cloning sites downstream to the CMV promoter. The sequence of BMP2 and TGFβ3 is shown in Table 1. The minicircle vectors were produced following the manufacturer’s instructions. ZYCY10P3S2T *Escherichia coli* cells were transformed with the parental vectors containing each growth factor. A single colony was obtained and grown for 2 h in 2 mL of Luria–Bertani broth (LB) with 500 μg/mL kanamycin at 30 °C. In a 1 L flask, 100 μL of the starter culture was inoculated into 200 mL of terrific broth (TB) and incubated at 30 °C with shaking at 200 rpm for 15 h. The induction medium, consisting of 200 mL LB, 4% 1N NaOH, and 200 μL of 20% L-arabinose was added to the TB bacterial culture. The mixture was incubated for 5 h at 30 °C with shaking at 200 rpm. The bacterial cells were harvested and the plasmid DNA was extracted using NucleoBond Xtra plasmid purification kits (Macherey-Nagel, Duren, Germany). The resulting minicircle mock vector (mcMock) consisted of CMV-MCS-EF1-RFP-SV40-PolyA. The inserts encoded in the minicircles were confirmed by double digestion by XbaI and BamHI.

### 2.2. hiPSC Culture

All induced pluripotent stem cell (iPSC) lines used in this experiment (*n* = 3) were generated using umbilical cord blood mononuclear cells. Reprogramming and characterization were performed as previously described [4]. Cells were maintained in a vitronectin-coated dish (Thermo Fisher Scientific, Waltham, MA, USA) and media were changed daily with fresh Essential 8 medium (Thermo Fisher Scientific).

### 2.3. EB Generation and OG Induction

The maintained iPSCs were detached and 2 × 10^6^ cells were prepared. A 1:1 mixture of Essential 8 media and Aggrewell media (STEMCELL Technologies, Vancouver, Canada) was used to generate EBs in a 100 mm petri dish. Cells were incubated in the media mixture for 24 h in a 5% CO_2_ atmosphere at 37 °C. The media were changed daily with fresh E8 media for 3 days. On day 4, the EBs were transferred to E7 media consisting of Dulbecco’s Modified Eagle Medium/Nutrient Mixture F-12 (DMEM/F-12, Thermo Fisher Scientific), 7.5% NaHCO_3_ (Thermo Fisher Scientific), 64 μg/mL ascorbic acid 2-phosphate (Sigma Aldrich, St. Louis, MO, USA), 14 ng/mL sodium selenite (Sigma Aldrich), 10.7 μg/mL transferrin (Sigma Aldrich), 20 μg/mL insulin (Thermo Fisher Scientific), and 2 ng/mL TGFβ1 (Peprotech, Rocky Hill, NJ, USA). EBs were maintained for an additional 3 days. Gelatin-coated dished were prepared for outgrowth (OG) cell induction. Culture dishes were coated with 0.1% gelatin (Sigma Aldrich) overnight at 37 °C. EBs were harvested and resuspended in OG induction media consisting of Dulbecco’s Modified Eagle Medium (DMEM, Thermo Fisher Scientific), 20% fetal bovine serum (FBS, Thermo Fisher Scientific) and 1% penicillin/streptomycin (Thermo Fisher Scientific). EBs were counted and 50–70 EBs per cm^2^ were seeded onto a gelatin-coated dish. OG cells were induced from the attached EBs for 3 days in 5% CO_2_ at 37 °C. Next, cells were detached and the remaining EB clumps were removed using a 40 μm cell strainer (BD Technologies, Franklin Lakes, NJ, USA). Single OG cells were harvested and plated onto a new gelatin-coated dish ((1–5) × 10^4^ cell per cm^2^). Cells were used for up to 3 passages.

### 2.4. Minicircle Transfection

HEK293T cells or OG cells were detached and 4.5 × 10^4^ cells per cm^2^ were prepared for transfection. OG cells were seeded onto a gelatin-coated plate. On the day before transfection, culture media were changed to DMEM without serum and antibiotics. Cells were transfected with the minicircle plasmids using the Lipofectamine 2000 reagent (Thermo Fisher Scientific), following the manufacturer’s instructions. Briefly, plasmid DNA and lipofectamine were mixed in Opti-MEM (Thermo Fisher Scientific) for 20 min. The DNA-lipid mixture was added to the culture media and incubated for 4 h in a 5% CO_2_ atmosphere at 37 °C. Media were changed into the OG induction media and incubated overnight. On the next day, the expression of red fluorescence protein (RFP) in the transfected OG cells was measured using fluorescence microscopy. The chondrogenic pellets were generated 72 h after transfection.

### 2.5. Enzyme-Linked Immunosorbent Assay

Cell sup from transfected HEK293T cells were harvested and transferred to a 96-well microtiter plate. The cultured sup was harvested 72 h after transfection. The plate was incubated at 4 °C overnight for coating. The next day, the culture sup was removed, and the plate was washed with PBS twice. The remaining protein-binding sites were blocked by adding a blocking buffer consisting of 5% non-fat dry milk containing phosphate-buffered saline (PBS) and incubated at room temperature (RT) for 2 h. The blocking buffer was removed, and the plate was washed with PBS twice. The primary antibodies (1:100; anti-BMP2 and anti-TGFβ3 antibody; ab6285 and ab15537, Abcam, Cambridge, UK) were incubated for 2 h at RT. Plates were washed and the anti-hIgG-HRP antibody (115-035-003 and 111-035-003, Jackson ImmunoResearch, West Grove, PA, USA) was applied to the wells and incubated for 1 h at RT. The plate was washed and then incubated with a 3,3′,5,5′-tetramethylbenzidine (TMB) substrate solution (eBioscience, San Diego, CA, USA) for 15 min. After applying the stop solution in each well, the absorbance was measured at 450 nm.

### 2.6. Osteogenic Differentiation

OG cells were seeded onto a gelatin-coated plate at 5 × 10^4^ cells/cm^2^. Cells were maintained in osteogenic differentiation media consisting of DMEM (Thermo Fisher Scientific) supplemented with 15% fetal bovine serum (FBS, Thermo Fisher Scientific), 50 μg/mL of ascorbate-2-phosphate (Sigma Aldrich), 10 nmol/L of dexamethasone (Sigma Aldrich), and 10 mmol/L of β-glycerophosphate (Sigma Aldrich) for 21 days, with media changes every other day. Osteogenesis was confirmed by alizarin red staining using a commercial kit (Sigma Aldrich).

### 2.7. Adipogenic Differentiation

OG cells were seeded onto a gelatin-coated plate at 5 × 10^4^ cells/cm^2^. Cells were maintained in adipogenesis differentiation media (Thermo Fisher Scientific) for 21 days with media changes every other day. Adipogenesis was confirmed by staining with oil red O (Sigma Aldrich).

### 2.8. Chondrogenic Differentiation Using Pellet Culture

The minicircle-transfected OG cells were counted and 3 × 10^5^ cells per pellet were prepared. Cells were harvested in a 15 mL conical tube and the media were changed into chondrogenic differentiation media (CDM) consisting of DMEM supplemented with a 1% knockout serum replacement (Thermo Fisher Scientific), 1× non-essential amino acids(Thermo Fisher Scientific), 1 mM l-glutamine(Thermo Fisher Scientific), 1% sodium pyruvate (Sigma Aldrich), 1% ITS+ Premix (BD Biosciences, Franklin Lakes, NJ, USA), 10^−7^ M dexamethasone (Sigma Aldrich), 50 mM ascorbic acid(Sigma Aldrich), 40 μg/mL L-proline(Sigma Aldrich), without additional recombinant growth factors. Cells resuspended in CDM were centrifuged at 750× *g* for 5 min. Generated pellets were maintained for 30 days in a 5% CO_2_ atmosphere at 37 °C and media were changed every 3 days. As a positive control, mcMock pellets were supplemented with 10 ng/mL recombinant human TGFβ3 (Peprotech) and 50 ng/mL recombinant human BMP2 (Peprotech), as described in our previous work [5].

### 2.9. Polymerase Chain Reaction

Harvested chondrogenic pellets were stored at −80 °C before use. Samples were snap-frozen with liquid nitrogen and ground using a pestle. Ground chondrogenic pellet samples were incubated with Trizol (Thermo Fisher Scientific) and the mRNA was extracted. The RevertAid^TM^ First Strand cDNA Synthesis kit (Thermo Fisher Scientific) was used to synthesize cDNA from the extracted RNA. Mean cycle threshold values from triplicate experiments were used to calculate the gene expression normalized to the expression of GAPDH. The primer sequences are shown in Table 2.

### 2.10. Ethics

All procedures involving animals were carried out in accordance with the Laboratory Animals Welfare Act, the Guide for the Care and Use of Laboratory Animals, and the Guidelines and Policies for Rodent Experimentation provided by the Institutional Animal Care and Use Committee of the College of Medicine of the Catholic University of Korea. This study protocol was approved by the Institutional Review Board of the Catholic University of Korea (CUMC-2016-0226-01).

### 2.11. Osteochondral Defect Model

Sprague Dawley rats were anaesthetized. An osteochondral defect (1.5 × 1.5 × 1.5 mm) was created using a microdrill on the articular cartilage of the trochlear groove of the distal femur. After 30 days of in vitro differentiation, chondrogenic pellets were placed in the defect (1 pellet per defect) (*n* = 5). The arthrotomy and skin were closed with interrupted nylon sutures. After 8 weeks, rats were sacrificed for gross and histological analysis.

### 2.12. Histological Analysis

Chondrogenic pellet samples or rat joint samples were washed with phosphate-buffered saline (PBS). The samples were fixed in 4% paraformaldehyde for 2 h at RT. Dehydration was performed with increasing sequential ethanol solutions. Additional clearing was done with sequential ethanol-xylene mixtures and the samples were infiltrated with paraffin overnight. Paraffin blocks were fixed and 7-μm sections were obtained using a microtome. Before staining the sections, the slides were placed in a 60 °C oven for at least 10 min. The slides were immediately deparaffinized using xylene. The slides were rehydrated with a decreasing sequential ethanol series and rinsed with running tap water for 1 min each. For alcian blue staining, the slides were incubated in 1% Alcian blue solution (Sigma Aldrich, St. Louis, MO, USA) for 30 min at RT. Slides were washed with running tap water and counterstained with a nuclear fast red solution. For safranin O staining, slides were stained with Weigert’s hematoxylin (Sigma Aldrich) for 1 min at RT. The slides were washed in running tap water for 10 min. The slides were stained with a 0.001% fast green solution (Sigma Aldrich) and 0.1% safranin O (Sigma Aldrich) solution for 5 min each. Toluidine blue staining was done by incubating the hydrated slides in a 0.04% toluidine blue (Sigma Aldrich) solution for 10 min. Slides were washed in running tap water and dried for 10 min until completely dry. After the staining process, the slides were dehydrated with an increasing sequential ethanol series. Ethanol was cleared with 2 cycles of 100% xylene and the slides were mounted with a VectaMount^TM^ Permanent Mounting Medium (Vector Laboratories, Burlingame, CA, USA).

### 2.13. Immunohistochemistry

The slides were placed in a 60 °C oven for 10 min and deparaffinized with 2 cycles of xylene. The slides were rehydrated and incubated in boiling citrate buffer (Sigma Aldrich) for antigen unmasking. After cooling the unmasked slides, endogenous peroxidase activity was blocked by treating the slides with 3% hydrogen peroxide (Sigma Aldrich). The slides were washed and blocked with tris-buffered saline (TBS) containing 1% bovine serum albumin (BSA). The primary antibodies against chondrogenic marker proteins were diluted in the blocking solution in the following ratios: collagen type I (1/200; ab34710, Abcam), collagen type II (1/200; ab34712, Abcam), and collagen type X (1:500; ab58632, Abcam). For the confirmation of pluripotency, the anti-c-Myc (1:250; ab32072, Abcam), and anti-TRA-1-60 (1:200; MAB4360, Merck Millipore, Burlington, MA, USA) antibodies were used. Osteogenic marker proteins were confirmed using the anti-osteocalcin (1:100; sc-30044, Santa Cruz Biotechnology, Dallas, TX, USA) and anti-RUNX2 (1:100; M-70, Santa Cruz Biotechnology) antibodies. The slides were incubated with the diluted primary antibody at 4 °C overnight. The next day, the slides were washed with TBS containing 0.1% Tween 20. The secondary antibodies (1/200; biotinylated goat anti-rabbit IgG antibody, BA-1000, and biotinylated horse anti-mouse IgG antibody, BA-2000, Vector Laboratories) were diluted in a blocking buffer which was treated for 40 min at RT. For fluorescence staining, the Alexa Fluor 488 goat anti-mouse IgG (H + L) antibody (1:400; A11029, Molecular Probes, Eugene, OR, USA) and Alexa Fluor 594 goat anti-rabbit IgG (H + L) antibody (1:400; A11037, Molecular Probes) were used. After washing out the secondary antibody, the slides were treated with Avidin-Biotin Complex (ABC) reagent drops (Vector Laboratories) for 30 min. A 3,3′-diaminobenzidine (DAB) solution (Vector Laboratories) followed and was incubated for 5 min. The slides were washed and counterstained with Mayer’s hematoxylin (Sigma Aldrich) for 1 min. The slides were dehydrated and cleared. The slides were mounted, and staining was confirmed under either a bright field or fluorescence microscope. The stained areas of the histological sections were quantified and measured using the ImageJ program.

### 2.14. Flow Cytometry

MSCs were harvested and stained with anti-human CD44 antibodies conjugated to BV421 (#562890, BD Biosciences, San Jose, CA, USA), anti-human CD73 antibodies conjugated to CD73 (#11-0739-42, eBioscience, Waltham, MA, USA), anti-human CD90 antibodies conjugated to PerCP-Cy5.5 (#561557, BD Biosciences), and anti-human CD105 antibodies conjugated to PE-Cyanine7 (#25-1057-42, eBioscience). Cells were than examined in an LSR Fortessa cell analyzer (BD Biosciences). The data were analyzed using the FlowJo 7.6.5 software (TreeStar Inc., Ashland, OR, USA).

### 2.15. Statistical Analysis

All experiments were repeated three or more times. The data are presented as the mean ± standard deviation. Statistical analysis was performed, and graphs were drawn using GraphPad Prism 5 (GraphPad). A t-test was applied to analyze non-parametric quantitative datasets, and the one-tailed *p*-value was calculated. Kruskal–Wallis one-way Anova, followed by Dunn’s multiple comparison test, was used for several analyses. Here, statistical significance is indicated as follows: * *p* < 0.05; ** *p* < 0.01; *** *p* < 0.001.

### 2.16. Ethics

This study was approved by the Institutional Review Board (IRB) of the Catholic University of Korea (IRB Number: KC13TISI0775). Written informed consent was obtained from all participants involved in this study.

## 3. Results

### 3.1. Generation of Minicircles Encoding Human Growth Factors

**Figure 1:**
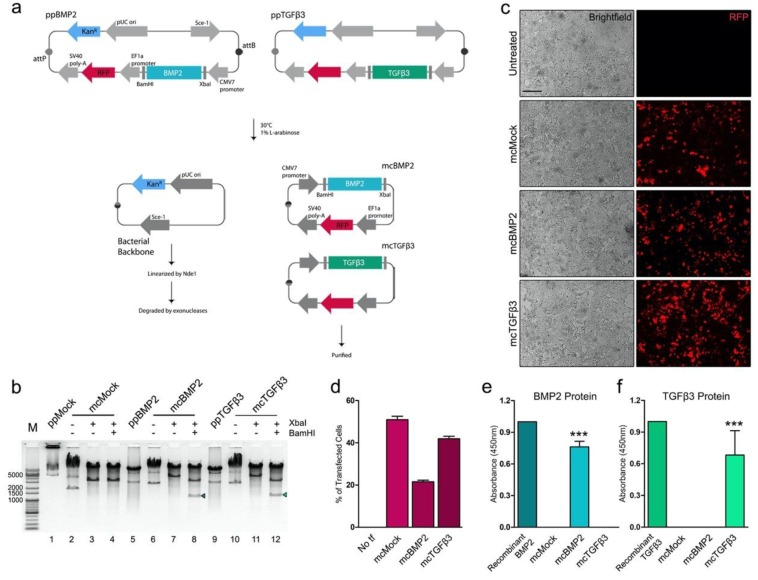
Generation of BMP2- and TGFβ3-encoding minicircles. (**a**) Scheme of minicircle generation process. (**b**) Gel electrophoresis of parental plasmid vectors (pp) and minicircle vectors (mc). The insert (arrow) of mcBMP2 has a size of 1194 bp and mcTGFβ3 has a size of 1236 bp. (**c**) Fluorescence microscopy image of transfected HEK293T cells. (**d**) Percentage of transfected HEK293T cells. (**e**) BMP2 protein expression in mcBMP2-transfected HEK293T sup. (**f**) TGFβ3 protein expression in mcTGFβ3-transfected HEK293T sup. Data presented as mean ± standard deviation from three independent sets of experiments. The scale bar represents 200 μm. *** *p* < 0.001 vs. mcMock indicates statistical significance. ppMock: parental mock vector; mcMock: minicircle mock vector; ppBMP2: parental BMP2-encoding vector; mcBMP2: minicircle BMP2-encoding vector; ppTGFβ3: parental TGFβ3-encoding vector; mcTGFβ3: minicircle TGFβ3-encoding vector; tf: transfection.

Human growth factor-encoding minicircle expression plasmid vectors were generated by synthesizing the codon-optimized cDNA of human BMP2 and TGFβ3. The sequence of propeptides and the active domain of each growth factor were fused for cloning to produce a more stable form of the synthesized growth factors (Table 1). The cDNA of each growth factor was sub-cloned into the parental plasmid vector downstream to the CMV promoter (Figure 1a). The insert sequences were cloned into the BamHI and XbaI restriction sites in the multiple cloning sites. The sizes of the BMP2-encoding minicircles (mcBMP2) were ~7.3 kb and TGFβ3-encoding minicircles (mcTGFβ3) had a size of approximately 7.5 kb. Successful cloning was confirmed by double digestion of the generated minicircles with BamHI and XbaI. A reduced size (~5 kb) of the growth factor-encoding minicircles was confirmed. The resulting fragment of BMP2 and TGFβ3 inserts was observed as a size of ~1.1 kb (Figure 1b). The insert of mcBMP2 has a size of 1,194 bp and mcTGFβ3 has a size of 1,236 bp.

The transfection efficacy of mcBMP2 and mcTGFβ3 was confirmed by transfecting HEK293T cells. The expression of red fluorescence protein was observed (Figure 1c). The cells transfected with the mock vector (mcMock) had the highest transfection efficacy. The expression of mcBMP2 was relatively lower than that of mcTGFβ3. The transfection efficacy of mcTGFβ3 was similar to that of mcMock (Figure 1d). The growth factor proteins secreted from the minicircles were detected from the supernatant of the transfected HEK293T cells. The absorbance of mcBMP2-transfected HEK293T cell supernatant was high, specifically, as much as the commercial recombinant protein BMP2 (concentrated 0.1 ng/mL) (Figure 1e). The protein expression of mcTGFβ3 was relatively low compared to that of the commercial recombinant protein TGFβ3 (concentrated 0.1 ng/mL), however, the level was similar to that of mcBMP2 (Figure 1f). Compared to the supernatant of mcMock, the absorbance was significantly detected in the supernatant of mcTGFβ3-transfected HEK293T cells. Based on these results, we confirmed the successful cloning of growth factor-encoding minicircles and detected the growth factors secreted from these minicircle vectors.

### 3.2. Chondrogenesis with Minicircles Using Human iPSC-Derived OG Cells

**Figure 2:**
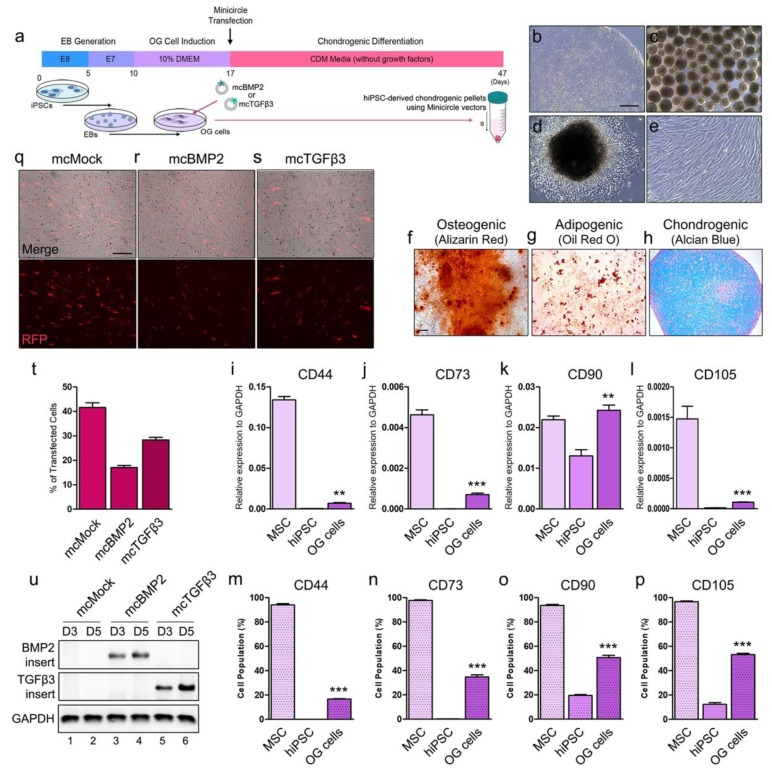
Chondrogenesis using minicircle-transfected hiPSC-derived OG cells. (**a**) Scheme of chondrogenic differentiation process from hiPSCs. Minicircles were transfected after OG cells were induced. (**b**) Morphology of the hiPSC colony. (**c**) Morphology of the generated EBs. (**d**) Image of outgrowth cells derived from EBs attached to a gelatin-coated culture dish. (**e**) Morphology of OG cells before transfection. (**f**) Alizarin red-stained osteogenic cells differentiated from OG cells. (**g**) Oil red O staining image of adipogenic cells differentiated from OG cells. (**h**) Chondrogenic pellet generated from OG cells stained with alcian blue. Relative gene expression of (**i**) CD44, (**j**) CD73, (**k**) CD90, and (**l**) CD105 in OG cells. Percentage of (**m**) CD44, (**n**) CD73, (**o**) CD90, and (**p**) CD105 positive cells. (**q**) Fluorescence microscopy of mcMock-transfected OG cells. (**r**) Fluorescence microscopy of mcBMP2-transfected OG cells. (**s**) Fluorescence microscopy of mcTGFβ3-transfected OG cells. (**t**) Percentage of OG cells transfected with each minicircle vectors. (**u**) Gel image of the PCR results against the insert of mcBMP2 and mcTGFβ3 in transfected OG cells. Data are presented as mean ± standard deviation from three independent sets of experiments. Scale bars represents 200 μm. ** *p* < 0.01 and *** *p* < 0.001 indicate statistical significance. EB: embryonic body; OG: outgrowth; CDM: chondrogenic differentiation media; RFP: red fluorescence protein; MSC: mesenchymal stem cell; hiPSC: human induced pluripotent stem cell; mcMock: minicircle mock vector-transfected outgrowth cells; mcBMP2: minicircle BMP2-encoding vector-transfected outgrowth cells; mcTGFβ3: minicircle TGFβ3-encoding vector-transfected outgrowth cells.

Here, hiPSCs were generated into EBs for OG induction. Mesenchymal-like OG cells were induced from the EBs for transfection. Minicircle vectors were transfected in OG cells one day before chondrogenic differentiation using pellet culture system. The chondrogenic pellets were maintained for 30 days without recombinant growth factor addition to observe the differentiation efficacy of mcBMP2- and mcTGFβ3-derived growth factors (Figure 2a). Stable hiPSCs with a compact colony were used for differentiation (Figure 2b). Cells were aggregated to generate EBs (Figure 2c). After attaching the EBs onto a gelatin-coated dish, fibroblast-like OG cells were induced (Figure 2d). After maintaining the OG cells for a week, cells showed a fibrotic morphology that is similar to mesenchymal stem cells. The cells showed a stable morphology after several days before differentiation (Figure 2e). MSCs are usually characterized by three-lineage differentiation (i.e., adipocytes, chondrocytes, and osteoblasts). OG cells successfully differentiated to an osteogenic lineage (Figure 2f). OG cells were also able to go through adipogenic differentiation as well (Figure 2g). The OG cells were able to generate chondrogenic pellets through differentiation with recombinant growth factors (Figure 2h). The gene expressions of MSC markers were examined in OG cells. Bone marrow-derived MSCs were used as a positive control. CD44 was increased in OG cells as compared to hiPSCs (Figure 2i). CD73 was increased in OG cells as well, however, it was significantly lower than that of MSCs (Figure 2j). Interestingly, the expression of CD90 was higher than that of MSCs (Figure 2k). Increased CD105 was also observed in OG cells (Figure 2l). MSC markers were all increased in OG cells, yet most of the markers were lower than that of the MSCs. The expression of MSC markers was also confirmed by FACS analysis (Figure 2m–p). The overall patterns were mostly similar to the genetic expression levels. While the marker expression was lower than that of MSCs, the expression was significantly increased compared to that of hiPSCs, which suggests that OG cells went through differentiation towards an MSC-like lineage. Transfection with mcMock in OG cells showed a high expression of RFP (Figure 2q). Similar to the results of HEK293T cells, mcBMP2 showed a relatively low expression of RFP in the OG cells (Figure 2r). Transfection with mcTGFβ3 showed similar results to that of the HEK293T cells (Figure 2s). We have confirmed that the overall transfection tendency of OG cells is similar to the tendency that shown by HEK293T cells (Figure 2t). The minicircle vector delivery was confirmed by PCR (Figure 2u). On days 3 and 5 after transfection, the expressions of mcBMP2 and mcTGFβ3 were confirmed in the transfected OG cells and the expression seemed to increase. Through these results, we have characterized the generated OG cells and confirmed the delivery of minicircle vectors in these cells.

### 3.3. Characterization of Minicircle-Based Chondrogenic Pellets

**Figure 3:**
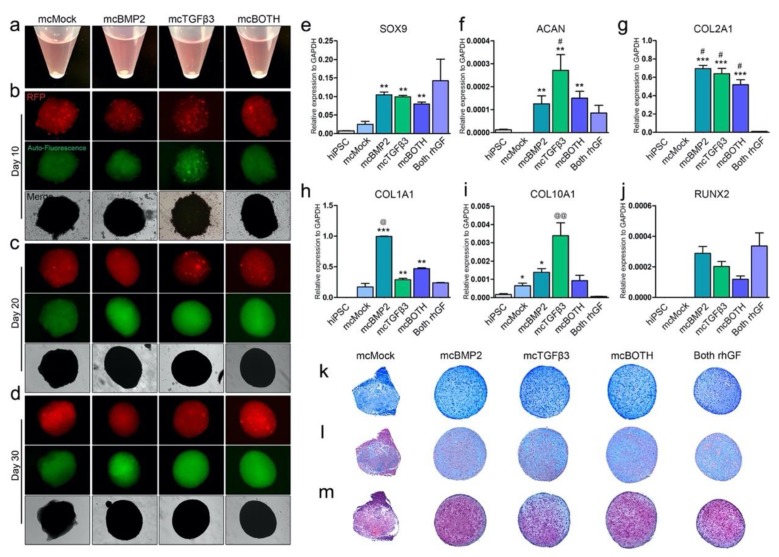
Minicircle expression in chondrogenic pellets and characterization of the generated pellets. (**a**) Morphology of pellets generated from hiPSC-derived OG cells on day 3. Fluorescence image of the chondrogenic pellets on (**b**) day 10, (**c**) 20, and (**d**) 30 of differentiation. Minicircle vectors were detected with RFP expression. GFP was detected to consider the auto-fluorescence of three-dimensional pellets. (**e**) Relative gene expression of SOX9 in chondrogenic pellets. (**f**) Relative gene expression of ACAN. (**g**) Relative gene expression of COL2A1. (**h**) Relative gene expression of COL1A1. (**i**) Relative gene expression of COL10A1. (**j**) Relative gene expression of the osteogenic marker, RUNX2. Data are presented as mean ± standard deviation from three independent sets of experiments. Each experiment analyzed 5 pellets per group. (**k**) Toluidine blue staining image of chondrogenic pellets. (**l**) Alcian blue staining of chondrogenic pellets for ECM detection. (**m**) Safranin O staining image of chondrogenic pellets. * *p* < 0.05; ** *p* < 0.01; *** *p* < 0.001 vs. mcMock (t-test), # *p* < 0.05 vs. mcMock, @ *p* < 0.05; @@ *p* < 0.01 vs. Both rhGF (one-way Anova) indicate significance. mcMock: minicircle mock vector-transfected outgrowth cells; mcBMP2: minicircle BMP2-encoding vector-transfected outgrowth cells; mcTGFβ3: minicircle TGFβ3-encoding vector-transfected outgrowth cells; mcBOTH: 1:1 mixture of mcBMP2- and mc TGFβ3-transfected outgrowth cells; Both rhGF: both recombinant human BMP2 and TGFβ3 growth factor protein-treated outgrowth cells.

OG cells were transfected with mcMock, mcBMP2, and mcTGFβ. After transfection, the cells were aggregated into pellets for differentiation. Cells transfected with mcBMP2 and mcTGFβ3 were mixed with the same portion and generated into pellets by centrifugation as well. The transfected cells formed a pellet after 3 days (Figure 3a). On day 10, the pellets maintained their morphology, and RFP expression was confirmed in the three-dimensional pellets (Figure 3b). The condensation of mcTGFβ3 pellets was later than others. Pellets were observed under a fluorescence microscope for 30 days. On day 20, the mcBMP2, mcTGFβ3, and mcBOTH pellets stayed in a condensed form (Figure 3c). The red fluorescence protein (RFP) expression of mcBMP2 and mcTGFβ3 was maintained up to 20 days but decreased when observed on day 30 (Figure 3d). Green fluorescence protein (GFP) was confirmed to discriminate autofluorescence. The RFP expression of mcMock was maintained until day 30 of differentiation.

The characteristics of the generated chondrogenic pellets were analyzed. The gene expression of chondrogenic markers was evaluated. Chondrogenic pellets generated using mcMock-transfected OG cells treated with human recombinant BMP2 and TGFβ3 were used as a positive control (Both rhGF). The early chondrogenic marker SOX9 was increased in the chondrogenic pellets differentiated with the minicircle-transfected cells and the expression level was almost similar to that of Both rhGF pellets (Figure 3e). The expression of ACAN was increased as higher as that of the chondrogenic pellets differentiated with the recombinant proteins (Figure 3f). SOX9 and ACAN were both significantly increased in mcBMP2, mcTGFβ3, and mcBOTH compared to mcMock. ACAN was most significantly increased in mcTGFβ3. COL2A1 is the gene responsible for the expression of collagen type II. The gene expression of COL2A1 was detected to be highest in all minicircle-transfected chondrogenic pellets except mcMock (Figure 3g). The expression was much higher than the pellets generated using the recombinant growth factors. The fibrotic marker COL1A1 and hypertrophic marker COL10A1 was mostly higher in the minicircle-transfected chondrogenic pellets (Figure 3h,i). Interestingly, mcBMP2 had the highest expression of COL1A1 and mcTGFβ3 had the highest expression of COL10A1. Yet, mcBOTH had the most moderate expression of all markers. The osteogenic marker RUNX2 was measured in pellets to confirm the osteogenicity of chondrogenic pellets (Figure 3j). All three conditions showed a lower expression of RUNX2 expression compared to the pellets differentiated by recombinant growth factors. Pellets generated with mcBOTH had the lowest expression of RUNX2. The gene expression of minicircle-based chondrogenic pellets was compared to that of the normal articular cartilage chondrocytes (Figure S1). Human articular chondrocytes maintained in monolayer culture (hChondrocyte), and pellets derived from articular chondrocytes maintained in CDM (hChondrocytePellet) were used. Chondrogenic pellets differentiated with minicircle-derived growth factors or recombinant growth factors had higher expression of SOX9 than human articular chondrocytes or the pellets generated using them (Figure S1a). While ACAN was significantly higher in articular chondrocytes (Figure S1b), COL2A1 expression was higher in pellets differentiated with growth factors-derived from minicircles (Figure S1c). Fibrotic marker COL1A1 was similar to that of monolayer-cultured articular chondrocytes; however, the expression levels increased in articular chondrocytes when maintained as a pellet in CDM (Figure S1d). Similar results were shown in hypertrophic markers, COL10A1 and RUNX2 (Figure S1e,f). The extracellular matrix (ECM) accumulation was detected by toluidine blue (Figure 3k), Alcian blue (Figure 3l), and safranin O (Figure 3m) staining. Taken together, we have confirmed the expression of minicircles in the iPSC-derived chondrogenic pellets, and the pellets have the characteristics of the chondrogenic lineage.

### 3.4. Further Analysis of Minicircle-Based Chondrogenic Pellets

**Figure 4:**
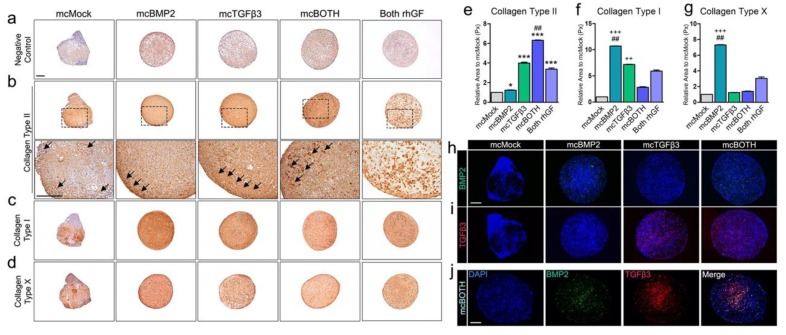
Further characterization of chondrogenic pellets generated with minicircles. (**a**) Image of antibody-negative control pellets used in immunohistology. (**b**) Image of pellets stained with collagen type II. The upper panel shows the 5x magnification image of each pellet and the lower panel shows the 200× magnification image. (**c**) Image of pellets stained with collagen type I. (**d**) Image of pellets stained with collagen type X. Quantification data of (**e**) collagen type II, (**f**) collagen type I, and (**g**) collagen type X. (**h**) Fluorescence image of pellets stained with the BMP2 antibody. (**i**) Fluorescence image of pellets stained with the TGFβ3 antibody. (**j**) Double staining image of the mcBOTH pellet. All scale bars represent 200 μm. * *p* < 0.05; ** *p* < 0.01; *** *p* < 0.001 vs. mcMock, ++ *p* < 0.01; +++ *p* < 0.001 vs. Both rhGF (t-test), ## *p* < 0.01 vs. mcMock (one-way Anova) indicate significance. mcMock: minicircle mock vector-transfected outgrowth cells; mcBMP2: minicircle BMP2-encoding vector-transfected outgrowth cells; mcTGFβ3: minicircle TGFβ3-encoding vector-transfected outgrowth cells; mcBOTH: 1:1 mixture of the mcBMP2- and mcTGFβ3-transfected outgrowth cells.

ECM production was detected in all pellets generated with minicircle vectors. For further analysis, the collagen type that consists of the produced ECM was analyzed. Collagen type II represents the hyaline cartilage and collagen type I represents the fibrotic cartilage in vivo. An antibody-negative control is shown in Figure 4a. The expression of collagen type II was highly detected in mcBMP2, mcTGFβ3, and mcBOTH pellets compared to mcMock (Figure 4b). While the high expression of collagen type II was confirmed in the surface of mcBMP2 pellets, lacunae-like morphologies were confirmed on the surface of mcTGFβ3 pellets (arrow). However, the highest staining intensity was confirmed in the mcBOTH pellets (arrow), which contain both mcBMP2 and mcTGFβ3 transfected cells (Figure 4e). Collagen type II was also detected in the mcMock pellets as well, yet, gland-like structures (arrow) inside the pellet suggests that the pellet has undergone random differentiation to a different lineage without the expression of growth factors. While all chondrogenic pellets had significantly higher expression of collagen type II compared to mcMock, mcBOTH pellets had the highest significance level (Figure 4e). The expression of collagen type I was also confirmed in pellets (Figure 4c). Collagen type I was relatively high in the mcBMP2 and mcTGFβ3 pellets, while the mcBOTH pellets had reduced expression (Figure 4f). Collagen type X is usually expressed in hypertrophic chondrocytes. The expression of collagen type X was also the highest in mcBMP2 pellets (Figure 4d,g). The expression of minicircle-derived growth factor proteins was detected in the pellets. Human BMP2 proteins were detected in mcBMP2 and mcBOTH by fluorescence staining (Figure 4h). The presence of human TGFβ3 proteins was also detected in mcTGFβ3 and mcBOTH pellets (Figure 4i). Double staining with both the anti-BMP2 and anti-TGFβ3 antibodies showed the distribution of cells transfected with mcBMP2 or mcTGFβ3 inside the mcBOTH chondrogenic pellets (Figure 4j). These results confirmed the expression of ECM proteins and also the expression of minicircle-derived human growth factors in the generated pellets.

### 3.5. In Vivo Transplantation of Chondrogenic Pellets in Osteochondral Rat Model

**Figure 5:**
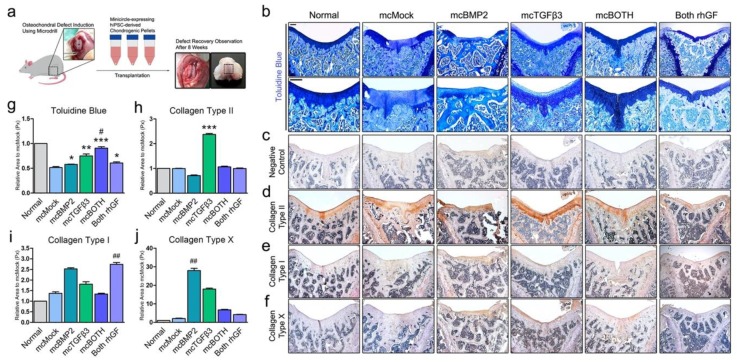
In vivo transplantation of minicircle-based chondrogenic pellets in the osteochondral defect rat model. (**a**) Scheme of defect induction and transplantation. (**b**) Image of pellet-transplanted joints stained with toluidine blue. The upper panel shows the 50× magnification images and the lower panel shows a 100× magnification images of the defect. (**c**) Image of antibody-negative control joint sections. Image of joints stained with (**d**) collagen type II, (**e**) collagen type I, and (**f**) collagen type X. (**g**) Quantification data of the toluidine blue stained area. (**h**) Quantification data of collagen type II. (**i**) Quantification data of collagen type I. (**j**) Quantification data of collagen type X. The scale bar represents 200 μm. * *p* < 0.05; ** *p* < 0.01; *** *p* < 0.001 vs. mcMock (t-test), # *p* < 0.05; ## *p* < 0.01 vs. normal (one-way Anova) indicate significance. mcMock: minicircle mock vector-transfected outgrowth cells; mcBMP2: minicircle BMP2-encoding vector-transfected outgrowth cells; mcTGFβ3: minicircle TGFβ3-encoding vector-transfected outgrowth cells; mcBOTH: 1:1 mixture of mcBMP2- and mc TGFβ3-transfected outgrowth cells; Both rhGF: both recombinant human BMP2 and TGFβ3 growth factor protein-treated outgrowth cells.

To confirm the recovery ability of minicircle-transfected chondrogenic pellets, we transplanted the pellets in the defect of an osteochondral rat model (Figure 5a). Defects were induced using a microdrill and chondrogenic pellets (on day 30) were placed in the defect. Rats were maintained for 8 weeks and sacrificed for further analysis. The recovery was evaluated by several staining methods. Toluidine blue staining showed that defects treated with minicircle-based chondrogenic pellets were healed (Figure 5b). The defect in joints implanted with mcMock pellets; however, had a relatively low accumulation of ECM proteins compared to the other groups (Figure 5g). The staining intensity of mcBoth was similar to that of the normal joint tissue. The protein types accumulated in the healed ECM matrix were further examined. The antibody-negative control is shown in Figure 5c. Collagen type II was highly expressed in joints implanted with mcTGFβ3 (Figure 5d). The expression was significantly highest in mcTGFβ3 pellet implanted joints compared to the other groups (Figure 5h). The expression of collagen type I was detected in the implanted joints (Figure 5e). Collagen I expression was reduced in mcBOTH pellet implanted joints when compared to that in BMP2 pellet implanted joints (Figure 5i); however, the highest staining intensity was shown in Both rhGF. The expression of collagen type X was also confirmed in the implanted joints (Figure 5f). Collagen type X had the highest expression in mcBMP2 pellet implanted joints (Figure 5j). Through these results, we have confirmed that minicircle-transfected chondrogenic pellets are able to heal osteochondral defects under in vivo conditions and the ECM protein types consisting the implants were analyzed.

### 3.6. Further Analysis of Implanted Chondrogenic Pellets

**Figure 6:**
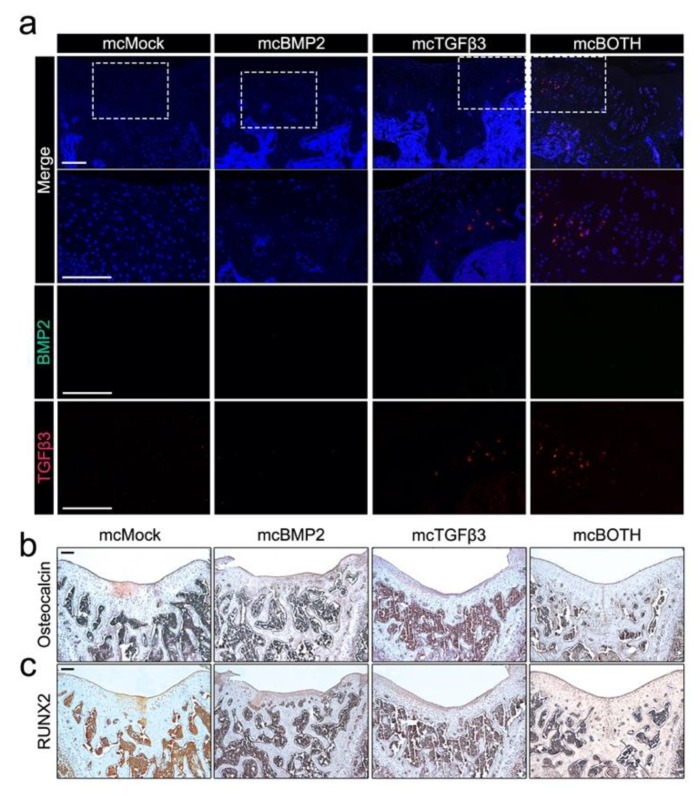
Further analysis of minicircle-based chondrogenic pellets transplanted in the osteochondral defect rat model. (**a**) Human BMP2 and TGFβ3 expression confirmation in pellet-implanted joints. Images show the 100× and 200× magnification image. (**b**) Image of pellet-transplanted joints stained with the osteocalcin antibody. (**c**) Image of joints stained with the RUNX2 antibody. Images show the 5× magnification image. All scale bars represent 200 μm. mcMock: minicircle mock vector-transfected outgrowth cells; mcBMP2: minicircle BMP2-encoding vector-transfected outgrowth cells; mcTGFβ3: minicircle TGFβ3-encoding vector-transfected outgrowth cells; mcBOTH: 1:1 mixture of mcBMP2- and mc TGFβ3-transfected outgrowth cells.

The expression of human BMP2 and TGFβ3 was confirmed in the implanted chondrogenic pellets using the BMP2 and TGFβ3 antibodies (Figure 6a). Growth factor expression was not confirmed in mcMock- and mcBMP2-implanted groups. In mcTGFβ3- and mcBOTH-implanted tissues, human TGFβ3 was confirmed by the RFP expression; however, the expression of human BMP2 was not shown in all samples.

It is crucial for the implants to maintain their chondrogenic characteristics rather than the osteogenic characteristics. To confirm the osteogenicity of the implants, we stained the samples with the osteocalcin and RUNX2 antibody, which are commonly used markers of osteogenesis (Figure 6b and c). Joints implanted with mcMock pellets showed positive staining of both markers, while other implants had relatively low expression. Through these results, we confirmed the possibility of remaining minicircle expression in the implants. Additionally, the data suggest low osteogenicity of the minicircle-based chondrogenic pellets after in vivo transplantation.

### 3.7. Further Analysis of Pluripotency and Tumorigenicity in Chondrogenic Pellets In Vitro and In Vivo

**Figure 7:**
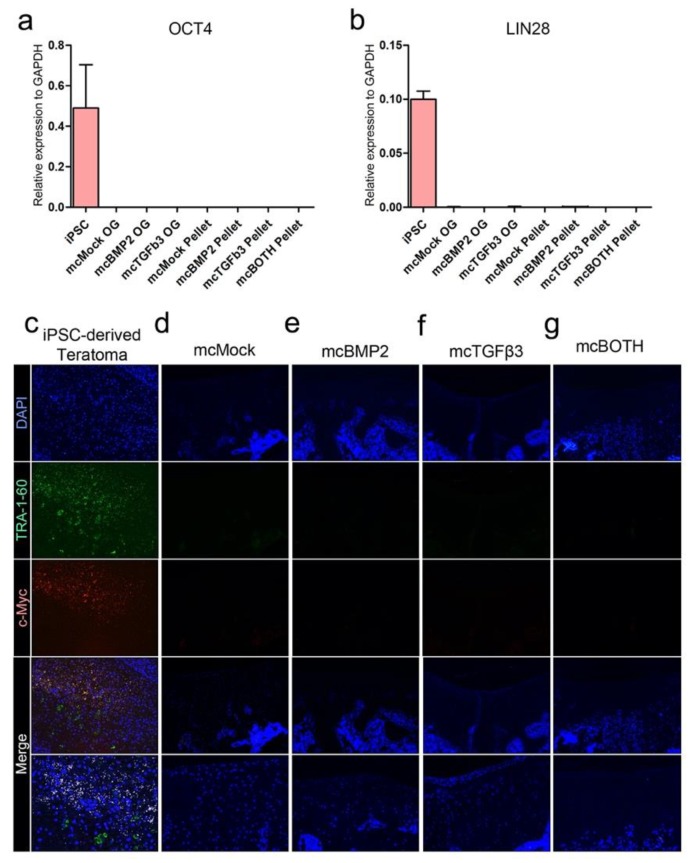
Confirmation of pluripotent markers in cells and tissue samples. (**a**) Relative gene expression of OCT4. (**b**) Relative gene expression of LIN28 in cells and pellets. (**c**) Fluorescence image of iPSC-derived teratoma tissues stained with the TRA-1-60 and c-Myc antibody. Fluorescence image of (**d**) mcMock, (**e**) mcBMP2, (**f**) mcTGFβ3, and (**g**) mcBOTH. Images show the 200× magnification image. Scale bar represents 200 μm. mcMock: minicircle mock vector-transfected outgrowth cells; mcBMP2: minicircle BMP2-encoding vector-transfected outgrowth cells; mcTGFβ3: minicircle TGFβ3-encoding vector-transfected outgrowth cells; mcBOTH: 1:1 mixture of mcBMP2- and mc TGFβ3-transfected outgrowth cells.

Since the chondrogenic pellets originate from hiPSC, we confirmed the impact of minicircle vectors on the expression of pluripotent and tumorigenic markers. The genetic levels of OCT4 were confirmed in transfected OG cells, and the pellets generated from these cells (Figure 7a). Compared to iPSCs, all of the cells did not show any expression of OCT4. LIN28 is a crucial marker of hiPSCs. The expression of LIN28 was also removed from the OG cells and the pellets (Figure 7b). The pluripotency and tumorigenic markers were confirmed in the in vivo implanted joints as well. Teratoma tissues derived from hiPSCs were used as a positive control (Figure 7c). While TRA-1-60 is a significant marker for hiPSC, it was absent in the joint tissues implanted with the generated chondrogenic pellets (Figure 7d–g). A representative tumorigenic marker, c-Myc, was also absent in the joint tissues, while it can be seen in the teratoma tissue. These data confirmed that pluripotency or tumorigenicity has been removed from the cells and the regenerated cartilage tissue, suggesting the safety of the minicircle-transfected OG cells and the implanted chondrogenic pellets generated using these cells.

## 4. Discussion

Low cellularity and avascularity limits cartilage recovery. The current option for cartilage recovery using cell-based therapy is the use of chondrocytes or MSCs. However, autologous chondrocytes and MSCs lose their original characteristics after several passages under in vitro conditions [29,30]. Dedifferentiated cells result in fibrocartilage after differentiation or transplantation. This challenge must be overcome for better cartilage recovery using cell-based therapy.

Human iPSCs are stem cells generated from adult somatic cells obtained from donors. The identical immunity with the donor makes hiPSCs able to avoid immune rejection. Also, hiPSCs can be expanded limitlessly in vitro. Therefore, they are a possible candidate as a next-generation material for cell-based therapy. The use of iPSCs in cartilage regeneration and recovery can suggest options for cell-based therapy to treat cartilage defects.

Non-viral gene delivery is a subject that should be solved for future application. Minicircles are supercoiled DNA vectors lacking the bacterial backbone sequence (i.e., the origin of replication, selection marker gene, and CpG motifs) [25,31]. The bacterial backbone in commercial plasmid vectors can induce immune reactions in cells [32]. The spreading of the antibiotic resistance gene can be unsafe for in vivo use. With robust construction and improved safety, minicircle vectors have been considered as a possible agent for DNA vaccination that can actually be used in clinics [33].

Here, we have shown the combination of minicircle technology and hiPSCs. Minicircle vectors encoding human BMP2 and TGFβ3 were successfully generated by cloning. OG cells induced from iPSC-derived EBs were transfected and showed a similar transfection efficacy to that of HEK293T cells. Chondrogenic pellets differentiated using growth factor proteins secreted from minicircles went through chondrogenesis in vitro and induced regeneration when transplanted in osteochondral defect rat joints. Minicircle transfected chondrogenic pellets showed an increase of ECM in the defected cartilage.

The delivery of mcBMP2 and mcTGFβ3 in hiPSC-derived OG cells successfully led to chondrogenesis. BMP2 and TGFβ3 have been reported to induce chondrogenic differentiation. BMP2 has previously restored and enhanced the chondrogenic potential of expanded chondrocytes under in vitro conditions [34]. TGFβ3 delivery was essential for neocartilage formation and increased the proliferation of MSCs [35]. Co-stimulation with both BMP2 and TGFβ3 has resulted in improved chondrogenesis compared to the standard one growth factor-based method. BMP2 synergistically enhances the effect of TGFβ3 [17]. In this study, we induced chondrogenesis by transfecting mcBMP2 and mcTGFβ3 into EB-derived OG cells. Separately transfected OG cells were mixed to confirm the effect on co-stimulation of BMP2 and TGFβ3. Co-stimulation with both growth factors showed a similar expression of SOX9 and COL2A1 compared to that of the other two conditions (Figure 3). However, mcBOTH pellets showed low expression levels of COL1A1 and COL10A1, while maintaining moderate levels of ACAN and COL2A1 expression. Additionally, these pellets had the lowest expression of RUNX2. Previous reports have shown that co-stimulation with both growth factors had lower levels of bone gamma-carboxyglutamate protein (BGLAP, osteocalcin) compared to both MSCs treated with TGFβ3 or undifferentiated MSCs [14]. On the protein levels, co-stimulated pellet mcBOTH have shown the highest intensity of collagen type II staining (Figure 4e). These results correspond with the previous reports, suggesting that co-stimulation with both BMP2 and TGFβ3 presents an improved quality in in vitro chondrogenesis. Since this study is an attempt as a pilot study for minicircle-based chondrogeneis; however, several improvements are required to upgrade the differentiation quality of the minicircle-based chondrogenic pellets and also for further analysis. To increase the differentiation efficacy of the minicircle-transfected OG cells, a positive selection of transfected cells, or double transfection of both vectors can be beneficial for further differentiation. Finding the optimal ratio of vectors for co-transfection based on the growth factor production rate might suggest a new protocol to generate chondrogenic pellets with improved quality.

Outgrowth cells generated from hiPSC-derived EBs have been reported to have similar qualities to that of MSCs [5,36,37]. The relative expression of MSC markers (i.e., CD44, CD73, CD90, and CD105) was evaluated. OG cells had increased levels of CD44, CD73, and CD105 compared to that of the hiPSCs (Figure 2). The memory of hiPSCs has already been reported by various researchers. Blood-derived hiPSCs result in more hematopoietic colonies than hiPSCs generated from fibroblasts [38]. Under hematoendothelial differentiation conditions, hiPSCs derived from umbilical cord vein endothelial cells and endothelial progenitor cells show higher efficiency compared to fibroblast-derived hiPSCs [39]. This may suggest that differentiated cells from the hiPSCs reflect the characteristics of their origin cell source by “epigenetic memory”. The characterization of MSCs-derived from hiPSCs has been reported by several researchers. Kang and colleagues previously reported that MSCs generated from hiPSCs have adequate osteogenicity and chondrogenicity; however, relatively less adipogenicity [40]. There is still less evidence about low adipogenicity; yet, there are several other studies that report the low adipogenicity of hiPSC-derived MSCs and even ESC-derived MSCs [41,42,43]. Yet, OG cells generated using cord blood mononuclear cell-derived hiPSCs were able to differentiate into all three lineages that are required for MSC characterization (i.e., osteogenic, adipogenic, and chondrogenic differentiation).

MSCs are infamous for having a low transfection efficiency with non-viral delivery. Lipid-based non-viral transfection systems show low efficiency in bone marrow-derived MSCs [44]. These results also agree with the report of a transfection efficacy lower than 5% in adipose-derived MSCs using lipofectants [45]. Even with minicircles, we have previously reported low transfection in bone marrow-derived MSCs using chemical transfection reagents [26]. The transfection efficacy in MSCs was increased when using electroporation along with minicircle vectors. However, MSC-like OG cells derived from hiPSCs showed high transfection efficacy even with lipofectants. This can suggest hiPSCs as a solution for finding a non-viral gene modified cell source for gene and cell therapy. In our results, mcBMP2 showed relatively low transfection efficacy compared to mcMock or mcTGFβ3. However, the protein secreted from the minicircle did not correlate with the transfection efficacy. Even with low transfection efficacy, mcBMP2 secreted relatively high levels of growth factor protein than mcTGFβ3. This is thought to be caused by the working structure of the protein. TGFβ3 is reported to have low solubility at a physiological pH and form aggregates more easily than the other TGF isoforms [46]. The cDNA of BMP2 and TGFβ3 inserted in the minicircle vectors include the active domain of each growth factor. To improve our chondrogenesis protocol using minicircle vectors, further analysis of the secreted growth factors is required. The exact structure and detection method using other assays such as western blot should be tested for the optimal use of vectors and the more accurate quantification of secreted growth factors. Additionally, the time point for the highest secretion of growth factors should be measured to improve our study in the future. In our previous studies, we have confirmed that the expression of transfected minicircle vectors maintained for about 30 days [26]. Chondrogenic pellets maintained minicircle vector expression during the differentiation process that lasted for 30 days; however, the expression tends to decrease during differentiation (Figure 3a-d). When we have confirmed the growth factor secretion in the rat knee joint tissue, expression of human TGFβ3 was also detected after 8 weeks of transplantation (Figure 6a). This might suggest that the effect of minicircle vectors remain even after 30 days of transfection; yet, additional analysis is required to confirm if it is the effect of minicircle vectors in vivo.

The healing ability of chondrogenic pellets was confirmed in osteochondral defect rat models. Compared to mcTGFβ3, mcBMP2 had a relatively small amount of accumulated collagen type II, which is a representative ECM protein for hyaline cartilage (Figure 5h). However, mcBMP2 had the highest expression of collagen types I and X between the minicircle-based pellets (Figure 5i,j). The pellets generated using mcBMP2 also had the highest expression of COL1A1 (Figure 3h). BMP2 was used as an additional agent for chondrogenesis; however, its ultimate function is also related to bone generation. The high expression of fibrotic and hypertrophic markers is thought to be related to the calcification of cartilage, which can lead to osteogenesis in the regenerated chondrogenic tissue. While mcBOTH pellets had the most moderate quality with high ACAN and COL2A1 expression and low COL1A1 and COL10A1 expression, the search for the optimal ratio of cell mixture for mcBOTH pellet generation might be efficient to improve the quality of the chondrogenic pellets. As mentioned earlier, the selection of transfected cells might be required to improve the efficacy of chondrogenesis, which can lead to a higher expression of growth factor secretion in vitro. Also, the confirmation of the best ratio for mcBOTH pellet generation, rather than a 1:1 mixture of cells, can be useful to search for the perfect mixture that may improve chondrogenesis without increasing the expression of fibrotic and hypertrophic markers. Additionally, finding the optimal ratio of mcBMP2 and mcTGFβ3 minicircle vectors for co-transfection can suggest another method to control the secretion of human BMP2 proteins in culture.

In this study, we report the first study on the application of minicircle vector-derived growth factors in in vitro chondrogenesis using hiPSCs. We conclude here that the minicircle encoding human BMP2 and TGFβ3 induced chondrogenesis in hiPSC-derived OG cells. Differentiation using human growth factors secreted from minicircles suggests a new regeneration process that can offer in vitro tissue regeneration, especially in combination with hiPSCs. There are still several details that requires further analysis; however, this proof-of-concept study suggests a new strategy for future regenerative medicines for cartilage damage.

## Figures and Tables

**Table 1:** The sequence of human BMP2 and TGFβ3 inserts. The black text indicates the propeptide sequence and the colored text indicates the sequence of the active domain of each growth factor.

**Human BMP2**
CTCGTTCCCGAGCTTGGTCGGAGGAAGTTTGCGGCCGCGTCAAGCGGAAGGCCCAGTAGTCAGCCT AGCGACGAGGTCCTTTCTGAATTCGAGCTTCGGCTCCTGTCCATGTTTGGACTTAAACAGCGACCTA CGCCCAGCCGGGATGCCGTTGTACCGCCCTATATGCTCGATCTTTATCGAAGACATTCCGGTCAGCCA GGATCACCGGCTCCAGATCATAGACTTGAGCGCGCTGCCTCCCGGGCAAACACTGTGCGATCCTTTC ACCATGAGGAATCACTGGAAGAATTGCCAGAAACTTCAGGTAAGACTACGAGACGATTCTTTTTTAA TCTCTCATCCATTCCTACAGAAGAATTCATTACGTCTGCCGAGCTTCAGGTATTCAGAGAACAGATGC AAGATGCTTTGGGGAATAACAGCAGCTTTCACCATCGCATCAACATATACGAGATAATCAAACCCGC AACAGCCAACAGCAAATTTCCCGTAACGCGATTGCTGGATACGCGACTTGTGAACCAAAACGCTAG CAGATGGGAATCATTCGATGTGACGCCCGCGGTCATGAGATGGACCGCTCAGGGCCACGCGAATCA CGGCTTTGTTGTAGAGGTGGCACATCTTGAAGAGAAGCAAGGTGTCAGCAAAAGACATGTACGAAT AAGTCGATCACTCCATCAAGATGAACACTCATGGAGCCAAATAAGACCTCTCCTTGTGACATTCGGG CATGACGGAAAGGGTCACCCTCTTCACAAAAGGGAGAAGCGC**CAGGCGAAGCATAAACAGCGGA** **AACGCCTTAAGTCAAGTTGCAAACGCCATCCTTTGTACGTCGATTTCTCCGATGTTGGATGGAAT** **GATTGGATCGTAGCTCCTCCTGGATACCATGCCTTCTATTGCCATGGCGAGTGCCCGTTCCCTCTT** **GCGGATCATCTCAACAGTACCAATCATGCAATCGTGCAAACCCTTGTAAACAGCGTCAACTCCAA** **AATTCCCAAGGCTTGTTGCGTTCCTACTGAGCTGAGCGCCATAAGTATGCTGTACCTCGATGAAA** **ATGAAAAAGTTGTCCTGAAGAATTATCAAGATATGGTGGTAGAAGGTTGTGGATGTAGG**
**Human TGFβ3**
TTGTCCACCTGTACTACTTTGGATTTTGGTCACATAAAAAAAAAACGGGTCGAGGCAATCCGAGGGC AAATTCTCAGCAAACTGAGGCTTACATCACCCCCCGAACCGACCGTTATGACCCACGTACCATATCA GGTCTTGGCTCTGTATAACTCTACTCGCGAACTGCTTGAGGAGATGCATGGGGAAAGAGAGGAGGG TTGTACCCAAGAGAATACCGAAAGCGAGTACTATGCTAAGGAGATTCATAAATTCGATATGATTCAG GGTCTGGCAGAGCACAACGAGCTGGCAGTGTGTCCAAAAGGAATCACCTCAAAGGTGTTTCGCTTC AATGTATCCAGCGTCGAAAAGAATCGCACCAACCTCTTCCGAGCGGAGTTTAGGGTTCTTCGGGTAC CAAACCCTAGCTCAAAGCGAAATGAGCAACGCATTGAGTTGTTCCAGATACTTAGGCCGGATGAAC ACATTGCGAAGCAGAGGTATATAGGTGGTAAAAACCTCCCGACTCGGGGTACTGCGGAGTGGCTCTC ATTTGATGTCACCGACACAGTACGCGAATGGCTTCTGCGAAGAGAGAGCAATCTTGGACTTGAAATC AGTATCCACTGTCCTTGTCATACCTTCCAACCGAATGGAGATATACTGGAGAACATCCACGAGGTAAT GGAAATTAAGTTTAAAGGCGTGGACAACGAAGATGATCACGGTCGGGGTGATCTGGGACGACTGAA GAAACAAAAAGACCACCATAACCCGCATCTGATCCTTATGATGATCCCCCCGCATAGACTCGACAAC CCAGGTCAAGGCGGGCAGAGAAAGAAAAGA**GCTCTGGATACTAACTACTGTTTTAGGAATCTGG** **AAGAAAACTGCTGCGTACGACCCTTGTATATTGATTTTAGACAAGACCTCGGTTGGAAATGGGT** **CCACGAACCAAAGGGATACTATGCCAATTTCTGTAGCGGCCCTTGTCCCTACTTGAGGAGTGCC** **GACACTACACATTCTACTGTGCTCGGTTTGTATAACACCTTGAACCCAGAAGCTAGTGCATCTCC** **CTGCTGCGTTCCCCAGGATCTCGAACCCCTCACTATTTTGTATTACGTTGGTCGGACACCAAAAG** **TCGAACAACTTTCAAACATGGTCGTGAAGTCCTGTAAGTGCAGC**

**Table 2:** Primer sequences for PCR experiments.

Gene Name	Sequences (5′ → 3′)	Length (bp)
CD44	F: AAGGTGGAGCAAACACAACC	151
	R: AGCTTTTTCTTCTGCCCACA	
CD73	F: CCAATTCTGAGTGCAAACAT	315
	R: CCTCCCACCACGACGTCCAC	
CD90	F: CTAGTGGACCAGAGCCTTCG	236
	R: TGGAGTGCACACGTGTAGGT	
CD105	F: CACTAGCCAGGTCTCGAAGG	165
	R: CTGAGGACCAGAAGCACCTC	
BMP2 insert (Active domain)	F: CAGGCGAAGCATAAACAGCG	342
	R: CCTACATCCACAACCTTCTACC	
TGFβ3 insert (Active domain)	F: CTGCTGCGTACGACCCTTG	295
	R: GCTGCACTTACAGGACTTCACG	
SOX9	F: GACTTCCGCGACGTGGAC	99
	R: GTTGGGCGGCAGGTACTG	
ACAN	F: TCGAGGACAGCGAGGCC	85
	R: TCGAGGGTGTAGCGTGTAGAGA	
COL2A1	F: GGCAATAGCAGGTTCACGTACA	79
	R: CGATAACAGTCTTGCCCCACTTA	
COL1A1	F: TCTGCGACAACGGCAAGGTG	146
	R: GACGCCGGTGGTTTCTTGGT	
COL10A1	F: CAGGCATAAAAGGCCCAC	108
	R: GTGGACCAGGAGTACCTTGC	
RUNX2	F: CTCTACCACCCCGCTGTCTT	143
	R: CACCTGCCTGGCTCTTCTTAC	
OCT4	F: GACAGGGGGAGGGGAGGAGCTAGG	144
	R: CTTCCCTCCAACCAGTTGCCCCAAAC	
LIN28	F: GTTCGGCTTCCTGTCCAT	121
	R: CTGCCTCACCCTCCTTCA	
GAPDH	F: ACCCACTCCTCCACCTTTGA	101
	R: CTGTTGCTGTAGCCAAATTCGT	

## Supplementary Materials

The following are available online at https://www.mdpi.com/2073-4409/9/3/582/s1, Figure S1: Relative expression of chondrogenic markers compared to human articular chondrocytes.
