# Supplementary material for: Chondrogenic Differentiation from Induced Pluripotent Stem Cells Using Non-Viral Minicircle Vectors
Source: Cells. 2020 Mar 1;9(3):582. doi: 10.3390/cells9030582 (PMC7140457; doi:10.3390/cells9030582)
Supplement: Supplementary file 1 [file cells-09-00582-s001.pdf]

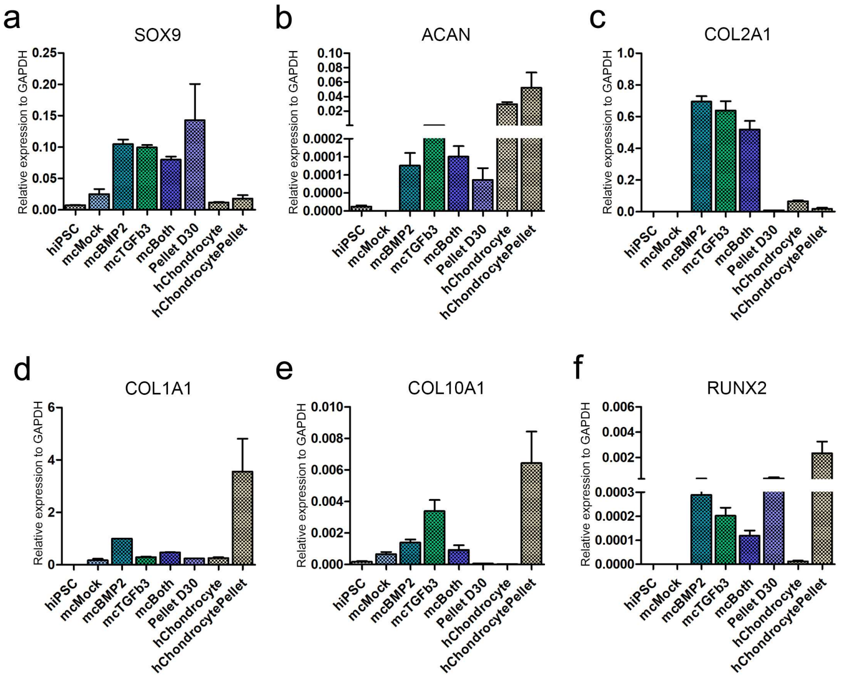

Figure S1. Relative expression of chondrogenic markers compared to human articular chondrocytes.
